# Bioremediation of Environmental Neonicotinoids Thiamethoxam and Clothianidin Contamination: Ecological Toxicity, Microbial Resources, Degradation Pathways, and Associated Enzymes

**DOI:** 10.3390/microorganisms14102190

**Published:** 2026-10-01

**Authors:** Shilei Sun

**Affiliations:** The Key Laboratory of Biotechnology for Medicinal Plants of Jiangsu Province, School of Life Sciences and Food Engineering, Jiangsu Normal University, Xuzhou 221116, China; sunshilei126com@126.com

**Keywords:** neonicotinoid insecticide, thiamethoxam, clothianidin, bioremediation, degradation mechanism

## Abstract

The chlorothiazolyl neonicotinoid insecticides thiamethoxam (THX) and clothianidin (CLO) are used widely in modern agriculture. However, their frequent application results in inevitable contamination of soil and water environments, posing potential ecological risks and threats to human health. Microbial remediation is an effective and environmentally friendly approach to addressing this issue. Some THX- and CLO-degrading microorganisms have been isolated and characterized, but their degradation mechanisms are not systematically reviewed. This review describes the environmental residues of THX and CLO, and their potential ecological toxicities toward terrestrial and aquatic ecosystems. Especially, the focus was paid to the degradation microorganisms and degradation pathways of THX and CLO, discussing the associated metabolic enzymes, including cytochrome P450 enzymes and aldehyde oxidases. This review deepens our understanding on microbial degradation mechanisms of THX and CLO, and provides theoretical and practical bases for the bioremediation of environmental THX and CLO contamination.

## 1. Introduction

Pesticides play a pivotal role in pest and disease control and crop yield improvement in agriculture. Neonicotinoid insecticides (NEOs), a substitute for older organophosphate, pyrethroid, and carbamate pesticides, have been recognized widely for their advantages of excellent efficiency and high selectivity [1,2]. They are used widely against sucking and chewing pests to protect yields of rice, wheat, maize, soybean, cotton, and potato [3]. In 2014, NEOs had a >25% share of the global pesticide market and been registered for use in >120 countries and regions [4]. Commercially used NEOs are divided into three categories based on their chemical structures: (i) chloropyridinyl NEOs, including imidacloprid (IMI), acetamiprid (ACE), thiacloprid (THI), and nitenpyram (NIT); (ii) chlorothiazolyl NEOs, including thiamethoxam (THX) and clothianidin (CLO); and (iii) tetrahydrofuranyl NEO dinotefuran (DIN) [5].

However, the excess and improper use of NEOs has given rise to environmental and ecological concerns. The application modes of NEOs include foliar sprays, stem applications, soil drenches, and seed treatment [6]. When NEOs are used on crops, only around 5% of the active ingredient is absorbed by the plants, and >90% migrates into the farmland soil, which significantly affects the health of soil ecosystems [7,8]. Residual NEOs in soil further migrate to surface and ground waters through precipitation, surface runoff, and leaching, generating threats to non-target aquatic organisms. Many studies have shown that the contamination of aquatic environments with NEOs decreases the abundance and biomass of insects, including Coleoptera, Diptera, and Ephemeroptera, and other aquatic fauna [9]. In addition, the extensive presence of NEOs in the food chain and living environments of humans gives rise to risks to human health. Research has indicated that exposure to the NEOs THX and IMI results in adverse health effects, including memory loss, neural tube defects, and autism spectrum disorder [10,11].

Various physicochemical strategies have been attempted to eliminate the environmental residue of NEOs, including membrane filtration, surface adsorption, and advanced oxidation processes [12]. However, these approaches bear certain deficiencies of incurring high implementation costs or generating secondary pollutants. In contrast, microbial remediation of NEOs is economical, environmentally friendly, and sustainable. The chlorothiazolyl NEOs THX and CLO are widely reported to persist in soil, water, and the food chain, generating significant environmental and ecological concerns [8]. However, the microorganisms reported to degrade THX and CLO are limited compared with other NEOs (such as the chloropyridinyls IMI, ACE, and THI). This review summarizes the reported environmental residues and toxicity of THX and CLO, followed by a comprehensive description of THX- and CLO-degrading microorganisms and degradation pathways, and eventually, the associated enzymes are discussed. The review presents a theoretical foundation for microbial remediation of environmental THX and CLO contamination.

## 2. Environmental Residues of THX and CLO

### 2.1. Soil Residues

THX and CLO are commonly used as seed treatments or applied directly to soil where they are absorbed by plant roots. North America presently accounts for the largest market share for seed treatments, where THX- or CLO-coated seeds applied to exceed 80% of maize seed [3,13]. There has been abundant research concerned with THX and CLO soil residues. Humann-Guilleminot et al. [14] measured the concentrations of five NEOs (IMI, CLO, THX, THI, and ACE) in soil in Switzerland and found that CLO was present in 77% of cultivated field soils and 46% of ecological focus area soils (annual, biennial, and perennial herbaceous plant species); THX was present in 27% of cultivated field soils and 6% of ecological focus area soils. NEOs have relatively long half-lives in soil, and the reported soil half-lives of THX and CLO are different, ranging from days to years. Goulson [6] reviewed estimated DT_50_ values (half-lives) in the soil of THX as 7–335 days, whereas the calculated half-life of CLO in soil ranges between 148 and 7000 days, exhibiting its recalcitrance. The persistence and fate of THX and CLO in soil are affected by many factors, including the inherent characteristics of the soil (composition, texture, and type) and external environmental factors (ultraviolet radiation, moisture, temperature, and pH). The organic matter and mineral clay content of soil affect the persistence of THX and CLO (high organic matter content of soil results in greater adsorption rates for THX and CLO). Dry soils with low temperatures and low exposure to ultraviolet radiation can result in the persistence and accumulation of THX and CLO [15,16].

The co-existence of THX and CLO in soils, especially the farmland soil, were widely reported in most countries, including China, the United Kingdom, the United States, Canada, Belize, and Germany [17], which have become a knotty problem. This is because on one side, the two pesticides have overlapping insecticidal spectra, usually alternately employed to combat a series of piercing pests, and on the other side, CLO is the primary degradation product of THX under natural environmental conditions.

### 2.2. Water Residue

THX and CLO have high water solubility (4100 mg/L for THX, and 340 mg/L for CLO at 20 °C, pH 7), high leaching potential, and low volatility [15]. Abundant studies have shown that THX and CLO extensively pollute various types of surface waters, including streams, wetlands, ditches, ponds, rivers, and drinking water [18]. In a study in Canada, 15 stream sites were sampled, and THX, CLO, and IMI exhibited detection rates >90% at more than half the sites sampled over the 3-year period 2012–2014 [19]. In another study, 65 sites in Minnesota, USA, representing rivers, streams, lakes, groundwater, and treated wastewater, were monitored, with CLO detection frequency reaching up to 100% in agricultural areas [20]. The contamination levels of THX and CLO differ in different areas of water bodies. The reported maximum concentrations of NEOs in surface waters are 6.90 µg/L for THX and 3.50 µg/L for CLO, which significantly exceed the ecological thresholds for NEO water concentrations (0.2 μg/L for short-term acute exposure and 0.035 μg/L for long-term chronic exposure), generating potential risks to aquatic non-target organisms [9,21].

## 3. Toxicity of THX and CLO

### 3.1. Effects on Terrestrial Organisms

After the application of THX and CLO in agricultural regions, most of the pesticide enters the soil environment, causing adverse effects on soil invertebrates. Earthworms exert a positive influence on maintaining soil health and account for approximately 60–80% of soil biomass [22]. Meng et al. [23] evaluated the toxicity effects of THX and CLO toward *Eisenia fetida* and found that THX and CLO inhibit the growth phenotype of earthworms and cause significant changes in antioxidant system and metabolic profiles. Ants inhabit soil and act as transporters and ecological engineers. Schläppi et al. [24] investigated the chronic exposure of black garden ant colonies (*Lasius niger*) to THX, and discovered that THX-exposed colonies had a decreased number of workers and larvae. THX and CLO function as systemic pesticides—once applied, they are absorbed by plants and translocated throughout the roots, stems, leaves, flowers, and fruits. Thus, pollinators are easy to suffer from their adverse effects. Acute oral exposure of bees of the species *Partamona helleri* to THX altered their behaviors, affected their traveled distance and meandering activity, and caused histopathological alterations in the midgut epithelium of workers [25]. Chronic exposure of honeybees to THX significantly altered ecdysone and juvenile hormone titers in both queen and drone larvae, impairing metamorphosis and reproductive development [26].

The decline in bird populations within agricultural areas has also been demonstrated to be associated with the extensive use of NEOs, particularly via consumption of NEO-treated seeds [27,28]. Lennon et al. [29] showed that, on sowing winter cereals, 32% of bird species observed in treated fields and 50% of individual birds were exposed to CLO post-sowing. In Ontario, Canada, MacDonald et al. [30] detected the carcasses of wild turkey that had consumed NEO-coated seeds and discovered detectable levels of THX and CLO in 22.5% of individuals. Such findings highlight the potential ecological risk of THX- and CLO-treated seeds to field birds in crop sowing periods.

NEOs are designed as high affinity agonists specially combing with insect nicotinic acetylcholine receptors (nAChRs), resulting in the paralysis and death of insects [31]. While NEOs have low affinity for mammalian nAChRs, increasing evidence shows sublethal effects on mammals. Issa et al. [32] investigated the toxicological effect of THX on adult male rats and found that exposure caused significant reproductive toxicity, with decreased testosterone levels, impaired sperm parameters, and histopathological damage to testicular tissue. In addition, continuous exposure of pregnant female rats to THX during the organogenesis period also caused reproductive toxicity through oxidative stress mechanisms, changes in fetal size and weight, visceral and skeletal malformations, and histological changes in tissues [33]. CLO is frequently detected in fruits and vegetables, so the potential exposure to humans has attracted great attention. Cheng et al. [34] evaluated the toxicity of CLO toward human Caco-2 cells and found that CLO induced cell apoptosis by inhibiting the metabolic enzyme CYP3A4, leading to oxidative stress and mitochondrial damage. Prisco et al. [35] investigated the influence of CLO on human immune signaling and discovered that CLO exposure significantly altered the transcription levels of various immune-related genes, resulting in potential immunotoxicity. THX- and CLO-induced cell toxicity can significantly impair the viability and function of reproductive, adipose, neural, immune, and epithelial cells. The underlying mechanisms involve oxidative stress, DNA damage, metabolic disturbances, mitochondrial dysfunction, endoplasmic reticulum stress response, and disruption of calcium homeostasis [36].

### 3.2. Effects on Aquatic Organisms

Due to the high mobility, THX and CLO are easily transferred from terrestrial to aquatic ecosystems. Mollusks are extremely sensitive to changes in the aquatic environment and are therefore excellent indicators of aquatic ecosystem health [37]. *Parreysia cylindrica* (freshwater mussels) were exposed to THX to study the histopathological effects; acute and chronic treatment with THX markedly affected the structure of the hepatopancreas, and an increase in structural damage to the hepatopancreas occurred with longer exposure to THX [38]. THX also showed toxic effects on aquatic arthropods. Decapod crustaceans like the Pacific white shrimp (Vannamei litopenaeus) show reduced growth performance and altered gut microecosystems due to THX-induced oxidative stress [39]. Miles et al. [40] investigated the toxicity of CLO toward 10 aquatic macroinvertebrates and three larval anuran species in the laboratory, and found LC_50_ values ranging from 0.002 to 1.2 ppm, which indicated potential detrimental effects of CLO on aquatic communities.

Some aquatic vertebrates also suffer from toxic effects of long-term exposure to THX and CLO. Fish are the primary high-quality protein source for humans from water bodies, and they play an important role in aquatic ecosystems as major inhabitants [41]. Exposure of adult zebrafish to THX altered their behavioral profile; caused tissue necrosis, erythrocyte infiltration, cloudy swelling, and other pathological changes in brain tissue; and affected the concentrations of acetylcholinesterase and cortisol, which was related to neurotoxicity [42]. Exposure of adult zebrafish to CLO led to increased activity of superoxide dismutase, lipid peroxidation, decreased catalase and glutathione *S*-transferase activities, and disturbed hepatic function [43]. Furthermore, increasing evidence shows the decline in amphibians correlated with the widespread use of NEOs [44]. Sublethal exposure of *Fejervarya cancrivora* (crab-eating frog) to CLO significantly impaired their growth and development, locomotor activity, predation efficiency, and affected their thyroid hormone regulation [45]. Fonseca Peña et al. [46] examined the toxicity of THX toward the tadpoles of three species of South American amphibians and discovered that THX significantly decreased their metamorphic success rate, affecting their survival in the wild.

## 4. THX- and CLO-Degrading Microorganisms

### 4.1. THX-Degrading Microorganisms

Microbial degradation of pesticides occurs via two main modes—catabolic metabolic degradation and co-metabolic degradation. The former uses the pesticide as the sole carbon and/or nitrogen source for growth; the latter requires extra nutrition in addition to the pesticide [47]. Bacteria are the main decomposer for microbial degradation of THX (Table 1). *Pseudomonas* sp. 1G was the first isolated strain that was reported to degrade THX. In the presence of 10 mmol/L glucose, it degraded about 70% of 50 mg/L THX in 14 days in microaerophilic conditions [48]. *Labrys portucalensis* F11 was able to remove 41% of 10.8 mg/L THX (provided as the sole carbon and nitrogen source) within 30 days, while periodic feeding with sodium acetate as an additional carbon source resulted in accelerated degradation of THX (>90% of THX removed in 3 days) [49]. Co-metabolism plays an important role in the microbial degradation of environmental pesticides, and the process is expedited with the supplement of readily utilizable carbon sources. However, for some kinds of microorganisms, they can degrade THX via catabolic metabolism without addition of extra carbon and/or nitrogen sources. *Bacillus aeromonas* IMBL 4.1 and *P. putida* IMBL 5.2 respectively degraded 45.28% and 38.23% of 50 mg/L THX in 15 days using THX as the sole source of carbon and energy, whereas, the degradation rates were reduced in the presence of easily metabolizable carbon and nitrogen sources, which might be because bacteria prefer to utilize easily available nutrients and exert their maximum potential only in nutrient-deficient conditions [50]. Similar phenomenon also occurred in *Bacillus Cereus* S1 degrading THX. A moderate increase in external carbon and nitrogen promotes the biodegradation rate of THX, whereas, when the C/N ratio exceeded 0.1%, the biodegradation rate decreased [51].

Plant growth-promoting rhizobacteria (PGPR) inhabit the rhizosphere and generate beneficial effects for plants through either direct or indirect mechanisms [52]. Much attention has recently been paid to bioremediation of contaminated soils using PGPR. *Ensifer* (*En*.) *adhaerens* TMX-23 is a plant growth-promoting rhizobacterial strain with the ability to release plant growth-promoting substances including indole-3-acetic acid, exopolysaccharides, ammonia, HCN, and siderophores. *En. adhaerens* TMX-23 degraded 37.5% of 200 mg/L THX within 25 days [53]. Other species of PGPR, such as *B. amyloliquefaciens* IN937a, *B. pumilus* SE34, and *B. subtilis* FZB24, can also degrade THX, with 11–22% degradation rates [54]. Therefore, those PGPR can be used as microbial inoculum applied in the agricultural field to promote the growth of field crops while remediating THX-contaminated soil.

The widespread use of THX on crops has inevitably led to the problem of pesticide residues in food. Endophytic bacteria inhabit the interior of plant tissues and possess abilities to eliminate contaminants inside their hosts [55]. An endophytic bacterial strain, *Enterobacter cloacae* TMX-6, was successfully inoculated into rice plants (*Oryza sativa* L.) and the highest concentration of THX in uninoculated rice samples was about 1.3-times more than that in inoculated rice plants, which indicated the potential of this strain for removal of THX residues from crops [56]. In recent years, joint plant–microbial remediation of pesticide pollution has been recognized as having enhanced degradation efficiency and environmental adaptive capacity compared with phyto or microbial remediation alone [57]. For example, around 33%, 77%, and 99% of THX were dissipated in the hydroponic medium inoculated with strain TMX-6 only, hydroponic medium with uninoculated rice only, and hydroponic medium with TMX-6-inoculated rice, respectively. The improved degradation ability of the plant–microbe combination might be attributable to the interaction between strain TMX-6 and the rice plants via communication mediated by signaling compounds [56]. This endophytic strain displays potential to be applied to eliminate THX residue in rice plants.

Algal systems are increasingly recognized for their dual advantages of low nutritional requirements and high pollutant degradation capacity [58]. Cyanobacteria, autotrophic prokaryotes, are widespread in various aquatic environments, including rivers, lakes, and oceans, and have been discovered to effectively use wastewater for growth, which facilitates pollutant removal [59,60]. *Nostoc* sp. PCC7120 was reported to remove 97% of 10 mg/L THX within 6 days, and THX promoted *Nostoc* cell growth [61]. The eukaryotic microalga *Scenedesmus* sp. TXH showed highly efficient degradation of, and high tolerance to, THX, with 100% removal of ≤40 mg/L THX and 97.5% removal of 60 mg/L THX within 12 days. Low concentrations of THX slightly promoted the growth of the microalga, while high-concentration THX severely inhibited microalgal growth [62]. The elimination efficiency of THX by this algal system was greater than that by reported bacteria, which shows the enormous potential of alga applied to THX remediation in polluted water bodies. Immobilized microorganism techniques are a sustainable solution for bioremediation of THX contamination and *Chryseobacterium* sp. H5 immobilized with polyvinyl alcohol/sodium alginate/biochar bead was used to enhance the biodegradation of THX. The results showed that the biodegradation rate of THX reached 68.03% and showed increased tolerance to extreme conditions [63].

### 4.2. CLO-Degrading Microorganisms

Compared with THX, the reported CLO-degrading microorganisms are limited (Table 1). *P. stutzeri* smk degraded 62% of 10 mg/L CLO within 14 days [64]. *Stutzerimonas* sp. SA1 and *Pseudomonas* sp. SA3, isolated from pesticide-contaminated agricultural soils, degrade various NEOs including THX, CLO, IMI, and ACE, with CLO (100 mg/L) degradation efficiencies of 77% and 53%, respectively, in 30 days [65].

Some fungi also possess NEO-degradation ability. White-rot fungi are widely applied to degrade various refractory organic pollutants, including polyaromatic hydrocarbons, endocrine-disrupting compounds, dyes, and pesticides [66,67]. The white-rot fungus *Phanerochaete* (*Ph*.) *sordida* degrades a series of NEOs, including ACE, THX, DIN, and NIT, to products with low neurological toxicity [68,69,70]. Mori et al. [55] reported that *Ph. sordida* degraded 37% of CLO within 20 days in nitrogen-limited liquid medium. White-rot fungi derive their bioremediation capabilities from specialized enzyme systems and their extracellular ligninolytic enzymes confer high toxin tolerance, which make them promising bioremediation agents for NEO contamination.

The microbial remediation ability of a single strain may be limited because of metabolic bottlenecks and restricted catabolic pathways [71]. Thus, in recent years, the use of microbial consortia to improve degradation efficiency and expand the degradation range of pollutants has been attempted. Microbial consortia can optimize metabolic burden distribution via quorum sensing mechanisms and facilitate biochemical functions unattainable in single-strain systems [5]. Wang et al. [72] constructed a mixed bacterial consortia SCAH, using four CLO-degrading strains: *Ochrobactrum anthropi*, *Acinetobacter johnsonii*, *Pseudomonas* sp., and *Stenotrophomonas maltophilia*. The individual CLO-degrading efficiencies of the four strains were 20.0–39.2% after 15 days of incubation in 500 mg/L mineral salt medium. In contrast, SCAH degraded 79.3% of 500 mg/L CLO in the same conditions. The consortia SCAH was also applied to CLO-contaminated soil in the presence or absence of additional nutrients, and the CLO was almost completely degraded after 45 days. Thus, SCAH has potential for use for in situ bioremediation of CLO contamination.

**Table 1:** The reported microorganisms that degrade THX and CLO.

NEOs	Microorganisms	Types	Comments	References
THX	*Pseudomonas* sp. 1G	Bacterium	70% degradation of 50 mg/L THX within 14 d	[48]
THX	*Labrys portucalensis* F11	Bacterium	41% removal of 10.8 mg/L THX within 30 d	[49]
THX	*Bacillus aeromonas* IMBL 4.1	Bacterium	45.28% degradation of 50 ug/mL THX within 15 d	[50]
THX	*Pseudomonas putida* IMBL 5.2	Bacterium	38.23% degradation of 50 ug/mL THX within 15 d	[50]
THX	*Bacillus cereus* S1	Bacterium	52.17% degradation of 90 mg/L THX within 216 h	[51]
THX	*Ensifer adhaerens* TMX-23	Bacterium	37.5% degradation of 200 mg/L THX within 25 d	[53]
THX	*B.* *amyloliquefaciens* IN937a	Bacterium	16–28% degradation of 2 mg/L THX within 72 h	[54]
THX	*B. pumilus* SE34	Bacterium	19–22 degradation of 2 mg/L THX within 72 h	[54]
THX	*B. subtilis* FZB24	Bacterium	11–14% degradation of 2 mg/L THX within 72 h	[54]
THX	*Enterobacter cloacae* TMX-6	Bacterium	33% degradation of 10 mg/L THX within 15 d	[56]
THX	*Nostoc* sp. PCC7120	Bacterium	97% removal of 10 mg/L THX within 6 d	[61]
THX	*Scenedesmus* sp. TXH	Algae	97.5% removal of 60 mg/L THX within 12 d	[62]
THX	*Chryseobacterium* sp H5	Bacterium	68.03% degradation of 14 mg/L THX within 120 h	[63]
THX	*Phanerochaete chrysosporium*	Fungus	98% removal of 10 mg/L THX within 25 d	[70]
CLO	*Phanerochaete sordida* YK-624	Fungus	37% degradation of CLO within 20d	[55]
CLO	*Pseudomonas stutzeri* smk	Bacterium	62% degradation of 10 mg/L CLO within 14d	[64]
CLO	*Pseudomonas* sp. SA3	Bacterium	53% degradation of 100 mg/L CLO within 30 d	[65]
CLO	*Stutzerimonas* sp. SA1	Bacterium	77% degradation of 100 mg/L CLO within 30 d	[65]
CLO	Consortia SCAH	Bacterium	79.3% degradation of 500 mg/L CLO within 15d	[72]

## 5. Microbial Degradation Pathways of THX and CLO

### 5.1. THX Degradation Pathways

**Figure 1:**
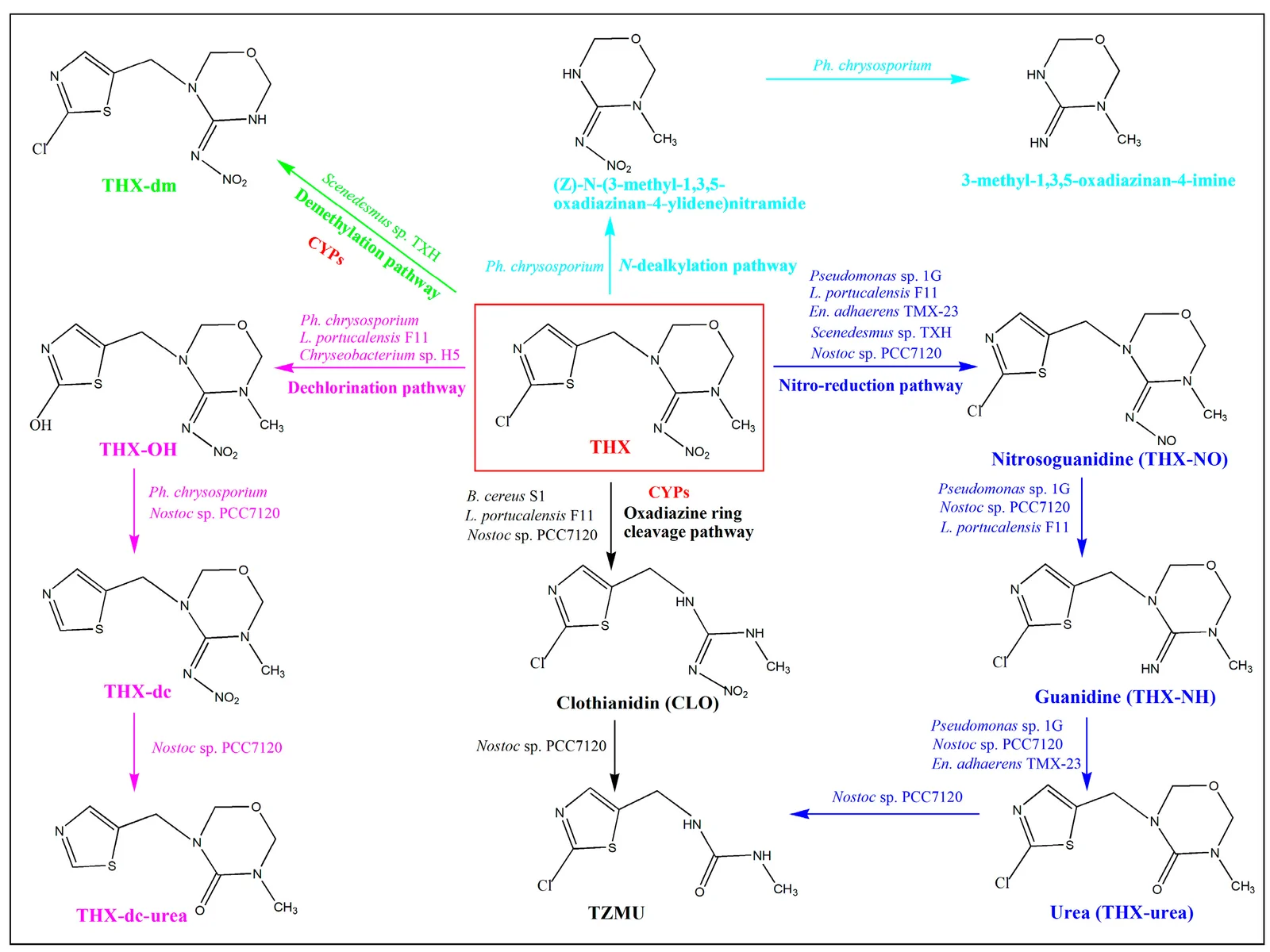
Proposed microbial degradation pathways of THX and associated enzymes. The primary degradation pathways of THX cover nitro-reduction, dechlorination, *N*-dealkylation, oxadiazine ring cleavage and demethylation pathways. The primary enzymes are cytochrome P450 enzymes (CYPs).

Multiple microbial degradation pathways of THX have been reported, which are confirmed by detection and identification of the corresponding degradation products (Figure 1). THX acts on the insect central nervous system and possesses high selectivity, which is attributed to its “magic nitro” (nitroimine, =N–NO_2_) group [73]. Biodegradation of THX often begins with the nitro moiety, via a nitro-reduction pathway. *Pseudomonas* sp. 1G reduces the nitro group of THX to a nitroso-guanidine (THX–NO) metabolite, which is further transformed to guanidine (THX–NH) and urea (THX–urea) derivatives [48]. A similar degradation pathway also occurred in *En. adhaerens* strain TMX-23 [53] and mammalian mice and goats [74]. The nitro-reduction pathway of THX is common, and suffered from particular focus, because the slight alterations of nitro group to guanidine might reverse the selective toxicity of THX toward insects over vertebrates [75]. Further toxicological studies of THX nitro-reduction metabolites is urgently required.

*Ph. chrysosporium* can degrade THX via dechlorination and *N*-dealkylation pathways. The dechlorination reaction begins with hydroxylating the substitution of the chlorine atom on the thiazole ring of THX, to generate THX–OH, followed by dehydration to form THX–dc, which is then converted to THX–dc–urea via nitro-reduction [70]. An analogous degradation pathway was reported in *Chryseobacterium* sp. H5 [63]. The *N*-dealkylation pathway stems from C–N cleavage between the 2-chlorothiazole ring and oxadiazine of THX, to produce (*Z*)-*N*-(3-methyl-1, 3, 5-oxadiazinan-4-ylidene)nitramide, which is further transformed to 3-methyl-1,3,5-oxadiazinan-4-imine via nitro degradation. The biotoxicity of THX and its metabolites was evaluated using *Escherichia coli*, *Microcystis aeruginosa*, rape, and cabbage. Compared with THX, the THX degradation products showed promoting effects on the growth of *E. coli* and *M. aeruginosa*, and on the germination rate index and sprout length of rape and cabbage seeds [70], which demonstrates that *Ph. chrysosporium* can be used effectively for the remediation of environmental THX contamination.

*Labrys portucalensis* F11 degrades THX via three different pathways: nitro-reduction, dechlorination, and oxadiazine ring cleavage [49]. The oxadiazine ring cleavage pathway is involved in the cleavage of THX oxadiazine ring to produce CLO, another common chlorothiazolyl NEO, and this pathway has been observed in mice, insects, spinach, tomato, soil [76] and the THX-degrading strain, *Bacillus cereus* S1 [51]. The generated CLO can be degraded further via nitro-reduction to form guanidine and urea metabolites of CLO. In *Scenedesmus* sp. TXH, CLO was not directly detected among the final THX degradation products, but the aminoguanidine metabolite of CLO was confirmed, indicating that CLO might occur as an easily degradable intermediate and undergo a quick degradation process. In addition, the demethylation product of THX (THX-dm) was also confirmed in *Scenedesmus* sp. TXH, produced via a demethylation pathway [62]. The toxicity of the THX degradation products of *L. portucalensis* F11 was assessed. Exposure to the final degradation products of THX produced by *L. portucalensis* F11 did not affect the seed germination, the bioluminescence of *Vibrio fischerim*, and the production of β-galactosidase by *E. coli*, which indicates that biodegradation of THX by *L. portucalensis* F11 is accompanied by a removal of toxicity [49].

### 5.2. CLO Degradation Pathways

**Figure 2:**
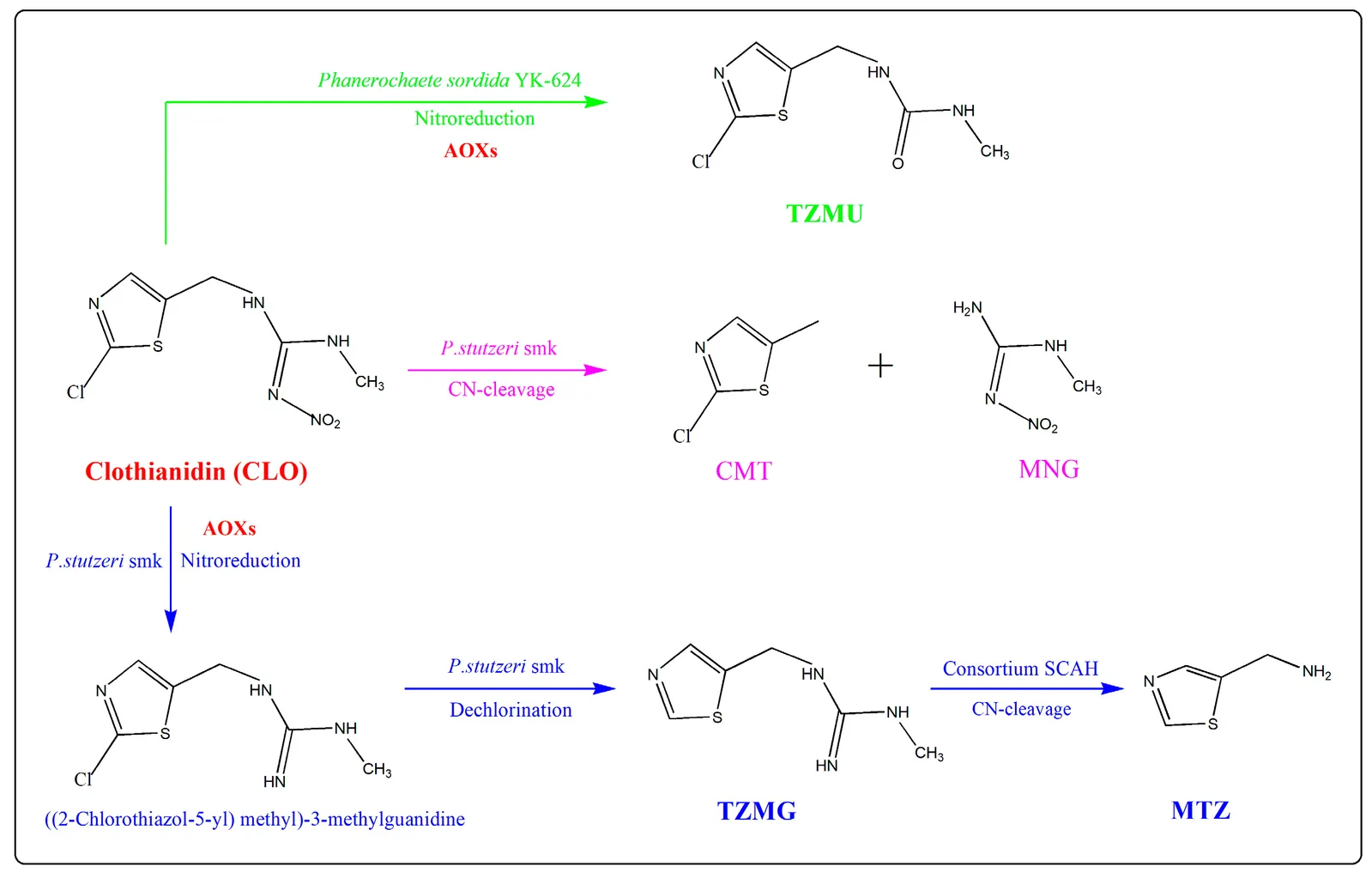
Proposed microbial degradation pathways of CLO and associated enzymes. The primary degradation pathways of CLO cover nitro-reduction and CN-cleavage pathways. The associated enzymes are aldehyde oxidases (AOXs).

CLO is the degradation product of THX via oxadiazine ring cleavage. They have the same pharmacophore nitroimine and hence, possess the analogous degradation pathways. However, in contrast, the reported microbial degradation pathways of CLO are scarce (Figure 2). The earliest report concerned the white-rot fungus *Ph. sordida*, which degraded CLO to *N*-(2-chlorothiazol-5-yl-methyl)-*N*-methylurea (TZMU) [55]. The metabolism of CLO has been investigated extensively in soil, animals, and plants. In mice and spinach, the crucial pharmacophore nitro of CLO is reduced to a nitrosoguanidine derivative, subsequently cleaved to generate guanidine and urea derivatives (TZMU) [76], which is similar to degradation of THX via the nitro-reduction pathway in *Pseudomonas* sp. 1G [48]. However, the relevant intermediates of this pathway were not detected in *Ph. sordida*. The effects of CLO and TZMU on the viability of the neuronal cell line Neuro2a were investigated, which indicated that TZMU lacks neurotoxicity [55].

Two CLO degradation pathways have been reported in *P. stutzeri* smk. One cleaves the C–N bonds between the thiazolyl methyl and guanidine moieties to form 2-chloro-5-methylthiazole (CMT) and (*Z*)-methyl-2-nitroguanidine (MNG), similar to the *N*-dealkylation pathway of THX degradation. The other involves denitrification and dehalogenation, resulting in the formation of ((2-chlorothiazol-5-yl)methyl)-3-methylguanidine and methyl-3-((thiazol-5-yl)methyl)guanidine (TZMG), respectively. Toxicity studies of CLO and its degradation products by *P. stutzeri* smk using alive mice showed significant reduction in the toxicity level of the degradation products to liver tissue [64]. Wang et al. [72] used the mixed bacterial consortia SCAH to degrade CLO and confirmed that the degradation products were TZMU and TZMG, which were in accordance with *Ph. sordida* [55] and *P. stutzeri* smk [64], respectively. The formed TZMG was further degraded to 5-amino-methlthiazol (MTZ) by cleavage of the C–N bond between the thiazolylmethyl and nitroguanidine moieties [72].

## 6. THX- and CLO-Metabolic Enzymes

### 6.1. Cytochrome P450 Enzymes

Cytochrome P450 enzymes (CYPs) are members of a versatile heme–mercaptide protein superfamily, which plays important roles in natural product biosynthesis and xenobiotic biodegradation [77]. White-rot fungi, a famous group of degraders in nature, are able to degrade various organic compounds using their extracellular ligninolytic enzymes and their intracellular CYP systems [78]. Wang et al. [79] used *Saccharomyces cerevisiae* to heterologously express *Ph. chrysosporium* CYPs and confirmed that CYP5147A3 can degrade ACE. Mori et al. [80] used CYP5147A3, and another isozyme CYP5037B3, to degrade the NEOs ACE, THI, IMI, and CLO. Both CYPs catalyzed cleavage of the chloropyridinyl moiety and the side-chain of ACE, THI, and IMI by *N*-dealkylation, resulting in the formation of 6-chloro-3-pyridinemethanol and respective side-chain fragments. The addition of cytochrome P450 inhibitors to the culture medium markedly decreased the degradation of CLO by *Ph. chrysosporium*. However, CYP5147A3 and CYP5037B3 did not degrade CLO, which indicates the presence of other CYPs involved in CLO degradation in *Ph. chrysosporium*.

Based on studies with specific recombinant enzymes, CYPs play a major role in pesticide metabolism [81]. Human CYPs CYP3A4, CYP2C19, and CYP2B6 degrade THX into CLO, and the generated CLO is further metabolized to desmethyl-CLO via demethylation by CYP3A4, CYP2C19, and CYP2A6. In addition, CYP2C19 degrades THX to desmethyl-THX [82]. In insects, CYP6EM1 confers the whitefly *Bemisia tabaci* with resistance to THX and directly metabolized 15.60% of THX in an in vitro metabolism assay [83]. Sattayawat et al. [84] heterologously expressed bee-derived CYP9Q1 in engineered *E. coli* BL21 (DE3) for THX detoxification. Whole cells heterologously expressing CYP9Q1 degraded THX to a desnitro metabolite, which highlighted the potential for developing probiotic or gut microbiome-based detoxification strategies to support the pesticide tolerance of bees.

### 6.2. Aldehyde Oxidases

Aldehyde oxidases (AOXs) belong to the molybdo–flavoenzyme superfamily, expressed in animal liver and play an important role in the metabolism of xenobiotics and drugs [85]. In mammals, nitro-reduction is a major metabolic pathway for NEO detoxification and AOX has been identified as the NEO nitroreductase [86]. Rabbit liver AOX is most active on CLO and desmethyl-THX, with DIN and IMI intermediate, and NIT and THX most slowly reduced [87]. In rabbit liver cytosol, AOX reduced CLO to nitrosoguanidine (CLO–NO) and aminoguanidine (CLO–NH_2_) metabolites. In contrast, human liver AOX converted CLO to CLO–NO, but not THX to THX–NO [82]. In bacterium strain *P. putida* KT2440, a three subunit AOX comprising a small subunit, a molybdenum-binding subunit, and a large subunit, was overexpressed in *E. coli*, and displayed nitroreductase ability to degrade IMI to nitroso IMI [88]. However, the large molecular weight of AOX presents challenges for its heterologous expression [89]. Microbial AOXs relevant to CLO and THX degradation have not been reported and require further study.

## 7. Conclusions and Prospects

The extensive and excessive use of THX and CLO in agriculture leads to abundant environmental residue and, thereby, generates potential threats to ecosystems and public health. Microbial remediation is considered a feasible approach to eliminate environmental THX and CLO contamination. Various microorganisms, including bacteria, fungi, and algae, have been isolated and applied in the remediation of THX and CLO contamination. This review summarizes the degrading microorganisms and degradation pathways of THX and CLO, and discusses the associated metabolic enzymes.

Some challenges remain to be addressed. First, the genes related to microbial degradation of THX and CLO, and their mechanisms of regulation of expression, remain hard to identify, which hampers further genetic modification to construct high-efficiency degrading strains. Thus, attention should be paid to elucidate the degradation-associated genes and their regulatory mechanisms in degrading strains via heterologous expression in *Saccharomyces cerevisiae* combined with genomics, transcriptomics, and proteomics analysis. Second, the toxicities of THX and CLO degradation products are detected in the form of mixtures, which results are inadequate for environmental risk assessments. Comprehensive risk assessments are needed for the individual degradation products of THX and CLO, especially the toxicological profiles of the nitro-reduction products. Third, as for the environmental THX and CLO pollution, plant growth-promoting bacteria, such as PGPR and endophytic bacteria, can be used as microbial inoculum independently, or develop integrated remediation strategies, such as joint plant–microbe remediation to accelerate elimination of environmental THX and CLO contamination. Fourth, the environmental co-contamination issue of THX and CLO is prominent and intractable. Utilizing different THX and CLO-degrading strains to develop engineered microbial consortia with an optimized metabolic flow, elevated degradation range, and enhanced synergistic interaction, is a promising solution to this problem.

## Data Availability

No new data were created or analyzed in this study. Data sharing is not applicable to this article.

## References

[1] Hladik M.L., Main A.R., Goulson D. (2018). Environmental risks and challenges associated with neonicotinoid insecticides. Environ. Sci. Technol..

[2] Ji K.M., Rong Y., Zhang Z.H., Chen Y.J., Hu R.X., Chu Y.C., Tang Y.Y., Qian L.J., Yang C. (2025). Neonicotinoids and human health: Environmental fate, toxicity mechanisms, and future directions. Pestic. Biochem. Physiol..

[3] Jeschke P., Nauen R., Schindler M., Elbert A. (2010). Overview of the status and global strategy for neonicotinoids. J. Agric. Food Chem..

[4] Zhang Y.Y., Zhu W.X., Wang Y., Li X.L., Lv J.X., Luo J.Y., Yang H.Y. (2024). Insight of neonicotinoid insecticides: Exploring exposure, mechanisms in non-target organisms, and removal technologies. Pharmacol. Res..

[5] Gautam P., Dubey S.K. (2023). Biodegradation of neonicotinoids: Current trends and future prospects. Curr. Pollut. Rep..

[6] Goulson D. (2013). An overview of the environmental risks posed by neonicotinoid insecticides. J. Appl. Ecol..

[7] Smalling K., Hladik M., Sanders C.J., Kuivila K.M. (2017). Leaching and sorption of neonicotinoid insecticides and fungicides from seed coatings. J. Environ. Sci. Health Part B.

[8] Wood T.J., Goulson D. (2017). The environmental risks of neonicotinoid pesticides: A review of the evidence post 2013. Environ. Sci. Pollut. Res..

[9] Morrissey C.A., Mineau P., Devries J.H., Sanchez-Bayo F., Liess M., Cavallaro M.C., Liber K. (2015). Neonicotinoid contamination of global surface waters and associated risk to aquatic invertebrates: A review. Environ. Int..

[10] Zhang Q., Li Z., Chang C.H., Lou J.L., Lu C. (2018). Potential human exposures to neonicotinoid insecticides: A review. Environ. Pollut..

[11] Zhang H., Zhang N., Zhou W., Zeng X., Wang X., Zhan M., Xu W., Huang Y., Lu L., Li Z. (2021). Profiles of neonicotinoid insecticides and their metabolites in paired saliva and periodontal blood samples in human from South China: Association with oxidative stress markers. Ecotoxicol. Environ. Saf..

[12] Mosavi S.S., Zare E.N., Behniafar H., Nezhad S.M., Neisiany R.E. (2024). Remediation of acetamiprid pesticide from contaminated water by antibacterial biosorbent based on carboxymethyl tragacanth-grafted-poly(3-aminophenol) decorated with ZnO@Fe_3_O_4_. Environ. Res..

[13] Alford A., Krupke C.H. (2017). Translocation of the neonicotinoid seed treatment clothianidin in maize. PLoS ONE.

[14] Humann-Guilleminot S., Binkowski Ł.J., Jenni L., Hilke G., Glauser G., Helfenstein F. (2019). A nation-wide survey of neonicotinoid insecticides in agricultural land with implications for agri-environment schemes. J. Appl. Ecol..

[15] Bonmatin J.-M., Giorio C., Girolami V., Goulson D., Kreutzweiser D.P., Krupke C., Liess M., Long E., Marzaro M., Mitchell E.A.D. (2015). Environmental fate and exposure; neonicotinoids and fipronil. Environ. Sci. Pollut. Res..

[16] Thompson D.A., Lehmler H.-J., Kolpin D.W., Hladik M.L., Vargo J.D., Schilling K.E., LeFevre G.H., Peeples T.L., Poch M.C., LaDuca L.E. (2020). A critical review on the potential impacts of neonicotinoid insecticide use: Current knowledge of environmental fate, toxicity, and implications for human health. Environ. Sci. Process. Impacts.

[17] Li F., Xiong W.P., Zhang C., Wang D.B., Zhou C.Y., Li W.B., Zeng G.M., Song B., Zeng Z.T. (2025). Neonicotinoid insecticides in non-target organisms: Occurrence, exposure, toxicity, and human health risks. J. Environ. Manag..

[18] Yang Q.L., Tang X.Q., He Y.Z., Wu X.Y., Yu X.B., Li Y.H., Wu Z.L. (2025). Assessing neonicotinoid pollution in aquatic ecosystems: A systematic review and bibliometric-content analysis. Comp. Biochem. Physiol. Part C.

[19] Struger J., Grabuski J., Cagampan S., Sverko E., McGoldrick D., Marvin C.H. (2017). Factors influencing the occurrence and distribution of neonicotinoid insecticides in surface waters of southern Ontario, Canada. Chemosphere.

[20] Berens M.J., Cape L.P.D., Arnold W.A. (2021). Neonicotinoid insecticides in surface water, groundwater, and wastewater across land-use gradients and potential effects. Environ. Toxicol. Chem..

[21] Evelsizer V., Skopec M. (2018). Pesticides, including neonicotinoids, in drained wetlands of Iowa’s prairie pothole region. Wetlands.

[22] Zhao W., Teng M., Zhang J., Wang K., Zhang J., Xu Y., Wang C. (2022). Insights into the mechanisms of organic pollutant toxicity to earthworms: Advances and perspectives. Environ. Pollut..

[23] Meng Z.Y., Yan Z.X., Yang R.X., Wu X.Y., Zhang X.L., Pan H., Bao X., Liu L., Chen X.J. (2024). Evaluating the toxicity effects of thiamethoxam and its main metabolite clothianidin to earthworms (*Eisenia fetida*) from the perspective of endogenous metabolites. Pestic. Biochem. Physiol..

[24] Schläppi D., Kettler N., Straub L., Glauser G., Neumann P. (2020). Long-term effects of neonicotinoid insecticides on ants. Commun. Biol..

[25] Motta J.V.O., Gomes D.S., Cruz1 J.P.P.O., Correia M.C.D., Bastos D.S.S., Oliveira L.L., Serrao J.E. (2025). Different concentrations of the insecticide thiamethoxam affects the midgut, behavior, and causes oxidative stress in the stingless bee *Partamona helleri* Friese, 1900 (Hymenoptera: Meliponini). Environ. Toxicol. Chem..

[26] Li M.J., He Q.B., Wu Y.F., Gao Q., Wang A.L., Xiao J.J., Liao M., Huang Y., Wang Y.H., Cao H.Q. (2025). Systemic Assessment of Chronic Toxicity of Thiamethoxam on Honeybees (*Apis mellifera*). Insects.

[27] Lennon R., Isaac J., Shore N.J.B., Peach R.F., Dunn W.J., Pereira J.C., Arnold M.G., Garthwaite K.E., Brown D. (2019). Using long-term datasets to assess the impacts of dietary exposure to neonicotinoids on farmland bird populations in England. PLoS ONE.

[28] Li Y.J., Miao R.Q., Khanna M. (2020). Neonicotinoids and decline in bird biodiversity in the United States. Nat. Sustain..

[29] Lennona R.J., Peach W.J., Dunn J.C., Shore R.F., Pereira M.G., Sleep D., Dodd S., Wheatley C.J., Arnold K.E., Brown C.D. (2020). From seeds to plasma: Confirmed exposure of multiple farmland bird species to clothianidin during sowing of winter cereals. Sci. Total Environ..

[30] MacDonald A.M., Jardine C.M., Thomas C.M., Nemeth P.J. (2018). Neonicotinoid detection in wild turkeys (*Meleagris gallopavo silvestris*) in Ontario, Canada. Environ. Sci. Pollut. Res..

[31] Houchat J.N., Cartereau A., Le Mauff A., Taillebois E., Thany S.H. (2020). An overview on the effect of neonicotinoid insecticides on mammalian cholinergic functions through the activation of neuronal nicotinic acetylcholine receptors. Int. J. Environ. Res. Public Health.

[32] Issa S.Y., Rahman S.M.A., Gaber Y.M., Soliman N.A.H. (2025). Toxicological impact of Thiamethoxam on adult male rats: Histopathological, biochemical, and oxidative DNA damage assessment. Toxicol. Rep..

[33] Hamed I.A., Sherif R.M., Shalaby A.A., Abdoslam O.E., He Q.Y., Li D.Y., Xu T., El-Sheikh E.A. (2025). Teratogenic effects of thiamethoxam on female rats and possible protective function of ascorbic acid. Open Vet. J..

[34] Cheng W.L., Zhang Z.H., Zhang Z.B., Zhao G.P., Wang Y.B. (2025). Clothianidin Exposure Induces Cell Apoptosis via Mitochondrial Oxidative Damage. Environ. Toxicol..

[35] Prisco G.D., Iannaccone M., Ianniello F., Ferrara R., Caprio E., Pennacchio F., Capparelli R. (2017). The neonicotinoid insecticide Clothianidin adversely afects immune signaling in a human cell line. Sci. Rep..

[36] Ma Y.Q., Wang Q.W. (2025). Neonicotinoid-Induced Cytotoxicity: Insights into Cellular Mechanisms and Health Risks. Toxics.

[37] Moraitis M.L., Tsikopoulou I., Geropoulos A., Dimitriou P.D., Papageorgiou N., Giannoulaki M., Valavanis V.D., Karakassis I. (2018). Molluscan indicator species and their potential use in ecological status assessment using species distribution modeling. Mar. Environ. Res..

[38] Patil A.G. (2019). Thiamethoxam induced histopathological alterations in hepatopancreas of freshwater bivalve Parreysia cylindrica. J. Entomol. Zool. Stud..

[39] Fu Z., Han F., Huang K., Zhang J., Qin J.G., Chen L., Li E. (2022). Combined toxic effects of thiamethoxam on intestinal flora, transcriptome and physiology of Pacific white shrimp Litopenaeus vannamei. Sci. Total Environ..

[40] Miles J.C., Hua J., Sepulveda M.S., Krupke C.H., Hoverman J.T. (2017). Effects of clothianidin on aquatic communities: Evaluating the impacts of lethal and sublethal exposure to neonicotinoids. PLoS ONE.

[41] Vajargah M.F., Yalsuyi A.M., Hedayati A., Faggio C. (2018). Histopathological lesions and toxicity in common carp (*Cyprinus carpio* L. 1758) induced by copper nanoparticles. Microsc. Res. Tech..

[42] Yang J.T., Guo C.S., Luo Y., Fan J.P., Wang W.M., Yin X.X., Xu J. (2023). Effect of thiamethoxam on the behavioral profile alteration and toxicity of adult zebrafish at environmentally relevant concentrations. Sci. Total Environ..

[43] Patil S., Roshan C.D. (2024). Effects of Clothianidin on Biochemical Parameters of Adult Zebrafish. J. Adv. Zool..

[44] Saka M., Tada N. (2021). Acute and chronic toxicity tests of systemic insecticides, four neonicotinoids and fipronil, using the tadpoles of the western clawed frog Silurana tropicalis. Chemosphere.

[45] Pilianto J., El-Ela A.A., Munawar A., Zhang X.F., Wang D., Soomro A.A., Ansari N.A., Zhou W.W., Zhu Z.R. (2026). Sublethal Clothianidin Exposure Impairs Development, Thyroid Hormones, Locomotion and Predation in *Fejervarya cancrivora* from Rice Paddy Ecosystems. Toxics.

[46] Fonseca Pena S.V.D., Natale G.S., Brodeur J.C. (2022). Toxicity of the neonicotinoid insecticides thiamethoxam and imidacloprid to tadpoles of three species of South American amphibians and effects of thiamethoxam on the metamorphosis of *Rhinella arenarum*. J. Toxicol. Environ. Health Part A.

[47] Pang S., Lin Z., Zhang W., Mishra S., Bhatt P., Chen S. (2020). Insights into the microbial degradation and biochemical mechanisms of neonicotinoids. Front. Microbiol..

[48] Pandey G., Dorrian S.J., Russell R.J., Oakeshott J.G. (2009). Biotransformation of the neonicotinoid insecticides imidacloprid and thiamethoxam by *Pseudomonas* sp. 1G. Biochem. Biophys. Res. Commun..

[49] Boufercha O., Monforte A.R., Boudemagh A., Ferreira A.C., Castro P.M.L., Moreira I.S. (2022). Biodegradation and Metabolic Pathway of the Neonicotinoid Insecticide Thiamethoxam by *Labrys portucalensis* F11. Int. J. Mol. Sci..

[50] Rana S., Jindal V., Mandal K., Kaur G., Gupta V.K. (2015). Thiamethoxam degradation by *Pseudomonas* and *Bacillus* strains isolated from agricultural soils. Environ. Monit. Assess..

[51] Xiang X.Z., Xie Y., Tian D., Chen Z.G., Yi X.H., Chen Z.Y., Huang M.Z. (2024). Microbial degradation mechanism and pathway of the insecticide thiamethoxam by isolated *Bacillus cereus* from activated sludge. Environ. Res..

[52] Sun S.L., Yang W.L., Fang W.W., Zhao Y.X., Guo L., Dai Y.J. (2018). The plant growth-promoting rhizobacterium *Variovorax boronicumulans* CGMCC 4969 regulates the level of indole-3-acetic acid synthesized from indole-3-acetonitrile. Appl. Environ. Microbiol..

[53] Zhou G.C., Wang Y., Zhai S., Ge F., Liu Z.H., Dai Y.J., Yuan S., Hou J.Y. (2013). Biodegradation of the neonicotinoid insecticide thiamethoxam by the nitrogen-fxing and plant-growth-promoting rhizobacterium *Ensifer adhaerens* strain TMX-23. Appl. Microbiol. Biotechnol..

[54] Myresiotis C.K., Vryzas Z., Papadopoulou-Mourkidou E. (2012). Biodegradation of soil-applied pesticides by selected strains of plant growth-promoting rhizobacteria (PGPR) and their effects on bacterial growth. Biodegradation.

[55] Mori T., Wang J., Tanaka Y., Nagai K., Kawagishi H., Hirai H. (2017). Bioremediation of the neonicotinoid insecticide clothianidin by the white-rot fungus *Phanerochaete sordida*. J. Hazard. Mater..

[56] Zhan H.L., Wan Q., Wang Y., Cheng J.F., Yu X.Y., Ge J. (2021). An endophytic bacterial strain, *Enterobacter cloacae* TMX-6, enhances the degradation of thiamethoxam in rice plants. Chemosphere.

[57] Supreeth M. (2022). Enhanced remediation of pollutants by microorganisms–plant combination. Int. J. Environ. Sci. Technol..

[58] Gondi R., Kavitha S., Kannah R.Y., Karthikeyan O.P., Kumar G., Tyagi V.K., Banu J.R. (2022). Algal-based system for removal of emerging pollutants from wastewater: A review. Bioresour. Technol..

[59] Nawaz T., Saud S., Gu L., Khan I., Fahad S., Zhou R. (2024). Cyanobacteria: Harnessing the power of microorganisms for plant growth promotion, stress alleviation, and phytoremediation in the era of sustainable agriculture. Plant Stress.

[60] Shahid A., Usman M., Atta Z., Musharraf S.G., Malik S., Elkamel A., Shahid M., Abdulhamid Alkhattabi N., Gull M., Mehmood M.A. (2021). Impact of wastewater cultivation on pollutant removal, biomass production, metabolite biosynthesis, and carbon dioxide fixation of newly isolated cyanobacteria in a multiproduct biorefinery paradigm. Bioresour. Technol..

[61] Zhu S.Y., Zhang J.L., Chen A.W., Chai Y.Z., Zeng J.H., Lu G., Bai M., Shao J.H., Peng L., Luo S. (2025). Newly discovered cyanobacteria *Nostoc* sp. PCC7120 for high efficiency biodegradation of thiamethoxam: Photosynthesis response, enzyme strategies, and molecular mechanisms. Bioresour. Technol..

[62] Quan L.H., Cheng Y.T., Wang J.P., Chen Y.L., Li D.T., Wang S.Q., Li B.L., Zhang Z.L., Yang L., Wu L. (2023). Efcient removal of thiamethoxam by freshwater microalgae *Scenedesmus* sp. TXH: Removal mechanism, metabolic degradation, and application. J. Environ. Manag..

[63] Xiang X.Z., Yi X.H., Zheng W.B., Li Y.Q., Zhang C., Wang X.Z., Chen Z.G., Huang M.Z., Ying G.G. (2023). Enhanced biodegradation of thiamethoxam with a novel polyvinyl alcohol (PVA)/sodium alginate (SA)/biochar immobilized *Chryseobacterium* sp. H5. J. Hazard. Mater..

[64] Parte S.G., Kharat A.S. (2019). Aerobic Degradation of Clothianidin to 2-Chloro-methyl Thiazole and Methyl 3-(Thiazole-yl) Methyl Guanidine Produced by *Pseudomonas stutzeri* smk. J. Environ. Public Health.

[65] Satheeshkumar A., Duraimurugan R., Velu P., Muthukumar B., Santhosh S., Devanesan S., AlSalhi M.S., Sathishkumar K., Rahman P.K., Rajasekar A. (2026). Biodegradation pathway and mechanism of new emerging pollutants neonicotinoids. J. Environ. Sci..

[66] Gao D., Du L., Yang J., Wu W.-M., Liang H. (2010). A critical review of the application of white rot fungus to environmental pollution control. Crit. Rev. Biotechnol..

[67] Wang J.Q., Majima N., Hirai H., Kawagishi H. (2012). Effective Removal of Endocrine-Disrupting Compounds by Lignin Peroxidase from the White-Rot Fungus *Phanerochaete sordida* YK-624. Curr. Microbiol..

[68] Wang J., Hirai H., Kawagishi H. (2012). Biotransformation of acetamiprid by the white-rot fungus *Phanerochaete sordida* YK-624. Appl. Microbiol. Biotechnol..

[69] Wang J., Tanaka Y., Ohno H., Jia J., Mori T., Xiao T., Yan B., Kawagishi H., Hirai H. (2019). Biotransformation and detoxification of the neonicotinoid insecticides nitenpyram and dinotefuran by *Phanerochaete sordida* YK-624. Environ. Pollut..

[70] Chen A.W., Li W.J., Zhang X.X., Shang C., Luo S., Cao R.Y., Jin D.D. (2021). Biodegradation and detoxification of neonicotinoid insecticide thiamethoxam by white-rot fungus *Phanerochaete chrysosporium*. J. Hazard. Mater..

[71] Lin Z.Q., Pang S.M., Zhou Z., Wu X.Z., Li J.Y., Huang Y.H., Chen S.H. (2022). Novel pathway of acephate degradation by the microbial consortium ZQ01 and its potential for environmental bioremediation. J. Hazard. Mater..

[72] Wang X., Xue L., Chang S., He X., Fan T., Wu J., Niu J., Eaneghmi B. (2019). Bioremediation and metabolism of clothianidin by mixed bacterial consortia enriched from contaminated soils in Chinese greenhouse. Process Biochem..

[73] Tomizawa M., Casida J.E. (2005). Neonicotinoid insecticide toxicology: Mechanisms of selective action. Annu. Rev. Pharmacol. Toxicol..

[74] Ford K.A., Casida J.E. (2006). Unique and common metabolites of thiamethoxam, clothianidin, and dinotefuran in mice. Chem. Res. Toxicol..

[75] Tomizawa M., Casida J.E. (2003). Selective toxicity of neonicotinoids attributable to specificity of insect and mammalian nicotinic receptors. Annu. Rev. Entomol..

[76] Casida J.E. (2011). Neonicotinoid Metabolism: Compounds, Substituents, Pathways, Enzymes, Organisms, and Relevance. J. Agric. Food Chem..

[77] Urlacher V.B., Girhard M. (2019). Cytochrome P450 monooxyge-nases in biotechnology and synthetic biology. Trends Biotechnol..

[78] Lin S., Wei J., Yang B., Zhang M., Zhuo R. (2022). Bioremediation of organic pollutants by white rot fungal cytochrome P450: The role and mechanism of CYP450 in biodegradation. Chemosphere.

[79] Wang J.Q., Ohno H., Ide Y., Ichinose H., Mori T., Kawagishi H., Hirai H. (2019). Identification of the cytochrome P450 involved in the degradation of neonicotinoid insecticide acetamiprid in *Phanerochaete chrysosporium*. J. Hazard. Mater..

[80] Mori T., Ohno H., Ichinose H., Kawagishi H., Hirai H. (2021). White-rot fungus *Phanerochaete chrysosporium* metabolizes chloropyridinyl-type neonicotinoid insecticides by an N-dealkylation reaction catalyzed by two cytochrome P450s. J. Hazard. Mater..

[81] Hodgson E. (2003). In vitro human phase I metabolism of xenobiotics I: Pesticides and related compounds used in agriculture and public health. J. Biochem. Mol. Toxicol..

[82] Shi X.Y., Dick R.A., Ford K.A., Casida J.E. (2009). Enzymes and inhibitors in neonicotinoid insecticide metabolism. J. Agric. Food Chem..

[83] Huang M., Gong P., Yin C., Yang J., Liu S., Fu B., Wei X., Liang J., Xue H., He C. (2025). Cytochrome P450 *CYP6EM1* confers resistance to thiamethoxam in the whitefly *Bemisia tabaci* (Hemiptera: Aleyrodidae) via detoxification metabolism. Pestic. Biochem. Physiol..

[84] Sattayawat P., Promubon K., Sripinta K., Chunguaongsuk W., Juntama C., Noirungsee N., Panya A., Disayathanoowat T. (2026). Proof-of-concept engineering of *Escherichia coli* expressing a bee-derived cytochrome P450 monooxygenase for thiamethoxam detoxification. J. Biol. Eng..

[85] Terao M., Garattini E., Romão M.J., Leimkühler S. (2020). Evolution, expression, and substrate specificities of aldehyde oxidase enzymes in eukaryotes. J. Biol. Chem..

[86] Dick R.A., Kanne D.B., Casida J.E. (2005). Identification of Aldehyde Oxidase as the Neonicotinoid Nitroreductase. Chem. Res. Toxicol..

[87] Dick R.A., Kanne D.B., Casida J.E. (2006). Substrate specificity of rabbit aldehyde oxidase for nitroguanidine and nitromethylene neonicotinoid insecticides. Chem. Res. Toxicol..

[88] Lu T.Q., Mao S.Y., Sun S.L., Yang W.L., Ge F., Dai Y.J. (2016). Regulation of hydroxylation and nitroreduction pathways during metabolism of the neonicotinoid insecticide imidacloprid by *Pseudomonas putida*. J. Agric. Food Chem..

[89] Swenson T.L., Casida J.E. (2013). Aldehyde oxidase importance in vivo in xenobiotic metabolism: Imidacloprid nitroreduction in mice. Toxicol. Sci..

